# Chloral as a Counter-Irritant

**Published:** 1879-01

**Authors:** 


Chloral as a Counter-Irritant.—Among the many uses to which chloral has been put, we have not met before with the following from the *Bulletin Therapeutique:*

"Made into a mass with gum tragacanth, spread on paper and applied to the skin, it will produce a blister without pain. Applied as a powder, on cotton, it causes a painful burning sensation. By the former method, a portion is absorbed and the patient falls asleep. Its action is not so uniform as cantharides, but is a mild vesicant, or an agreeable revulsive, the author quoted would commend such 'chloral paper' to physicians, the more so as it will keep for months without losing its activity, if well prepared."

